# Supplementary material for: Identification and functional analysis of non-coding regulatory small RNA FenSr3 in Bacillus amyloliquefaciens LPB-18
Source: PeerJ. 2023 May 15;11:e15236. doi: 10.7717/peerj.15236 (PMC10194069; doi:10.7717/peerj.15236)
Supplement: Supplemental Information 4 [file peerj-11-15236-s004.zip › KO/CK-vs-T1_map/map04973.html]

KEGG PATHWAY: Carbohydrate digestion and absorption - Reference pathway


|  |  |
| --- | --- |
| **Carbohydrate digestion and absorption - Reference pathway** |  |

[
Pathway menu
| Organism menu
| Pathway entry
| Show description
| User data mapping
]

Dietary carbohydrate in humans and omnivorous animals is a major nutrient. The carbohydrates that we ingest vary from the lactose in milk to complex carbohydrates. These carbohydrates are digested to monosaccharides, mostly glucose, galactose and fructose, prior to absorption in the small intestine. Glucose and galactose are initially transported into the enterocyte by SGLT1 located in the apical brush border membrane and then exit through the basolateral membrane by either GLUT2 or exocytosis. In a new model of intestinal glucose absorption, transport by SGLT1 induces rapid insertion and activation of GLUT2 in the brush border membrane by a PKC betaII-dependent mechanism. Moreover, trafficking of apical GLUT2 is rapidly up-regulated by glucose and artificial sweeteners, which act through T1R2 + T1R3/alpha-gustducin to activate PLC-beta2 and PKC-beta II. Fructose is transported separately by the brush border GLUT5 and then released out of the enterocyte into the blood by GLUT2.

|  |  |  |
| --- | --- | --- |
| Reference pathway | 184% 150% 122% 100% 82% 67% 55% | 图片下载 |
